# Nutritional Status and Immune Functions in Maintenance Hemodialysis Patients

**DOI:** 10.1155/MI/2006/20264

**Published:** 2006-02-21

**Authors:** Hayriye Sayarlioglu, Reha Erkoc, Cengiz Demir, Ekrem Dogan, Mehmet Sayarlioglu, Ahmet Faik Oner, Imdat Dilek

**Affiliations:** ^1^Division of Nephrology, Department of Internal Medicine, Medical Faculty, Yuzuncu Yil University, 65200 Van, Turkey; ^2^Division of Hematology, Department of Internal Medicine, Medical Faculty, Yuzuncu Yil University, 65200 Van, Turkey

## Abstract

Epidemiological studies suggest various kinds of immune dysregulation in hemodialysis (HD) patients. The aim of this study was to investigate the relationship between immune functions and nutritional status of HD patients. We studied 54 patients with ESRD on chronic HD, included 34 females and 20 males with mean age 46.6 ± 16.3 (18–77) years. We measured the height and dry weight of all patients. The BMI was calculated by dividing weight (kg) by height squared (m^2^). In all patients serum urea, creatinine, albumin, iron, cholesterol, triglyceride, CRP, IgG, IgM, IgA, CD4, CD8, CD19, CD16-56 lymphocytes were measured. Kt/V values were calculated according to DOQI guideline. In this study, a positive correlation between albumin, cholesterol, and triglyceride levels as nutritional parameters and immune functions in terms of total and subtype lymphocyte counts was observed. Further prospective studies are needed to determine the clinical importance of this finding and the appropriate means of measurement and effects of nutrition on immune function in hemodialysis patients.

## INTRODUCTION

End-stage renal failure induces a clinical state of immunodeficiency with higher incidence of infections and a higher mortality due to infectious complications compared with the normal population [1]. Epidemiological studies indicate that in chronic hemodialysis (HD) patients, bacterial and viral infection ranks second place in mortality and morbidity, behind cardiovascular disease. Several studies suggest different kinds of immune dysregulation in HD patients [2, 3]. Uremic toxins may cause defects in cell-mediated immunity. Clinical evidence of impaired lymphocyte, granulocyte, and monocyte functions, which progress during the development of uremic retention, has been reported. Malnutrition is an important problem in patients treated with chronic hemodialysis or peritoneal dialysis. It occurs in 40 to 70 percent of patients (depending upon the method used to measure nutritional status), with an increasing length of time on dialysis correlating with an increasing decline in nutritional parameters [4, 5]. The role of nutrition in improving survival in HD patients has been increasingly appreciated. Generally, nutrient deficiencies are associated with impaired immune responses. The aspects of immunity most consistently affected by malnutrition are cell-mediated immunity, phagocyte function, the complement system, secretory antibody, and cytokine production [6]. Previous data showed that delayed cutaneous hypersensitivity reactions are markedly depressed in mild and moderate malnutrition and the number of circulating CD4 lymphocytes is reduced [7]. In one study, it was shown that poor nutritional status might be a cause of impaired lymphocyte function in HD patients [8]. The aim of this study was to investigate the relationship between immune functions and nutritional status of HD patients.

## METHODS

We studied 54 patients with ESRD on chronic HD, included 34 females and 20 males with mean age 46.6 ± 16.3 (18–77) years. The mean time on HD was 3.6 ± 2.9 (1–15) years. We measured the height and dry weight of all patients. The BMI was calculated by dividing weight (kg) by height squared (m^2^). At the time of sampling, no patient had an active infection, ongoing inflammatory disease, diabetes mellitus, and immune disorders. Moreover, no patient was taking immunosuppressive drug. With respect to virological status, no patient was HIV positive. CRP values were under 5 mg/ml in all patients. All blood samples were drawn before the second dialysis session of the week. In all patients serum urea, creatinine, albumin, iron, cholesterol, triglyceride, and CRP via colorimetric method were measured. Serum concentrations of IgG, IgM, and IgA were measured by immunonephelometry (Dade Behring, Marburg, Germany). CD4, CD8, CD19, CD16-56 lymphocytes were counted using flow cytometry (Beckman-Coulter EPICS-XL, USA). Kt/V values were calculated according to DOQI guideline. Descriptive statistics and Pearson's correlation tests were performed.

## RESULTS

Descriptive data and laboratory parameters are shown in Table 1. Correlation analysis between immune and some other parameters is shown in Table 2.

Positive correlation of serum albumin levels with total lymphocyte count and CD4 count, positive correlation of serum cholesterol and triglyceride levels with total lymphocyte count and its subtypes, negative correlation of serum hemoglobin levels with CD4/CD8 ratio and negative correlation of serum ferritin with the number of CD8 (T-suppressor cell), CD19, and total lymphocyte count were observed.

Also Kt/V values were correlated with ferritin and hemoglobin values (resp, *r* = 0.44, *P* = .002, *r* = 0.36, *P* = .01). There were negative correlations between Kt/V values and serum triglyceride levels and BMI (resp, *r* = −0.302, *P* = .03, *r* = −0.372, *P* = .007).

## DISCUSSION

Malnutrition has an effect on immune parameters in HD patients. This increases the tendency to infections, and also fastens the atherosclerosis. These factors are the most important ones in determining the mortality and morbidity in HD patients. Pupim et al showed that nutritional status of chronic HD patients predicted mortality independent of concomitant presence or absence of inflammatory response [9].

Food-derived substances incorporated into the body via various routes modulate immune functions. Taking into consideration that malnutrition or calorie restriction causes reduced activity in immune functions, nutritional condition is indispensable for the development of the immune system. Generally nutrient deficiencies are associated with impaired immune responses. The aspects of immunity most consistently affected by malnutrition are cell-mediated immunity, phagocyte function, the complement system, secretory antibody, and cytokine production.

Chronic uremia is often accompanied by depressed cellular and humoral immunity. Actually, it is difficult to define whether the altered immune response is associated mainly with uremia rather than with the induction of malnutrition or the dialysis therapy. However, it is clear that malnutrition and uremia induce severe alterations in the host defense and specific immune systems if both diseases occur [10, 11], contributing to the high incidence of infection in dialysis patients [12].

We investigated the relationship between immune function and nutritional status of HD patients. Nutritional parameters of HD patients were evaluated with serum albumin, cholesterol, triglyceride, ferritin, iron, iron-binding capacity, and body mass index (BMI). In our study, serum cholesterol, albumin, and triglyceride levels were positively correlated with lymphocyte and lymphocyte subtypes. Mean lymphocyte counts of our patients were below normal (1398.3 ± 455.6), whereas immunoglobulin levels were normal.

Humoral immune responses remain apparently intact in situations of protein-energy malnutrition, when immunoglobulins and B cell number or function have been measured [13]. Wolfson et al in their study indicated that the typical patient undergoing maintenance hemodialysis showed evidence for wasting or malnutrition. The patients demonstrated abnormal parameters of nutritional status even though they all were clinically stable and had not experienced superimposed catabolic illnesses for at least 3 months before the study. In this study, lymphocyte transformation correlated directly with serum urea nitrogen and creatinine, as well as with serum albumin and midarm muscle circumference, suggesting that nutritional status may influence lymphocyte function in uremic patients [8]. Likewise, there was a positive correlation between albumin levels and lymphocyte counts and lymphocyte subgroups in our patients.

Lower cholesterol and albumin levels were associated with increased mortality in chronic hemodialysis patients [14]. Long-chain polyunsatured fatty acid (PUFA) in foods can modulate immune functions. Dietary *n*-3 PUFA alters the lipid composition of the cell membrane and regulates the function of immune cells. Antigen-presenting cells from mice and humans fed *n*-3 PUFA exhibited the capacity to suppress excessive activation of T cells [15, 16].

In our study, cholesterol and triglyceride levels were positively correlated with lymphocytes and their subgroups.

In one study, anthropometric measurements revealed an increased rate of malnutrition in common variable immunodeficiency patients, particularly in those with low CD4 and undetectable IgA [17]. In our patients, there is no correlation between BMI and lymphocytes and subgroups.

Effects of iron overload include decreased antibody-mediated and mitogen-stimulated phagocytosis by monocytes and macrophages, alterations in T-lymphocyte subsets, and modification of lymphocyte distribution in different compartments of the immune system. The poor ability of lymphocytes to sequester excess iron in ferritin may help explain the immune system abnormalities in iron-overloaded patients [18]. In our patients, negative correlations of serum ferritin with the number of CD8 (T-suppressor cell) and total lymphocyte count were observed. One most consider due to routine intravenous iron treatment in HD patients, ferritin level is not a nutrition parameter, but rather the result of the treatment.

Correlation between hemoglobin and CD4/CD8 ratio without any correlation for absolute numbers of CD4 and CD8 could not be explained and discussed; to our knowledge clinical importance of this condition is not known.

## CONCLUSION

In this study, a positive correlation between albumin, cholesterol, and triglyceride levels as nutritional parameters and immune functions in terms of total and subtype lymphocyte counts was observed. Further prospective studies are needed to determine the clinical importance of this finding and the appropriate means and effects of nutrition on immune function in hemodialysis patients.

## Figures and Tables

**Table 1:** Descriptive data for the study group for measured parameters.

	Mean	Std deviation	Normal range

Lymphocyte	1398.3	455.6	1500–4000/mm3
IgA (g/l)	2.2	0.9	0.7–4 g/l
IgM (g/l)	1.1	0.6	0.4–2.3 g/l
IgG (g/l)	12.8	4.2	7–16 g/l
CD3	945.7	412.8	
CD16	154.4	110.8	
CD19	114.1	60.9	
CD4	608.6	247,3	
CD8	337	155.3	
CD4/CD8	1.9	0.7	1.1–3
Kt/V	1.1	0.3	>1.2
CD4 (%)	43.5	8.5	35–65
CD8 (%)	24.2	6.6	20–35
CD19 (%)	8.1	3.8	2–15
CD 16 56 (%)	11	5.6	<10
Albumin (g/dl)	3.6	0.4	3–5.5
Hg (g/dl)	9.2	1.9	(12–16)
Urea (mg/dl)	167	48	14–45
Creatinine (mg/dl)	10	3.4	0.7–1.2
Iron (μ/dl)	82	53.7	37–157
Ferritin (ng/ml)	679	447	28–365
Cholesterol (mg/dl)	139.5	35	0–200
Triglyceride (mg/dl)	164.7	72.7	0–200

**Table 2:** Correlation between some nutritional or certain parameters and immune parameters. Upper values are Pearson's correlation coefficients; the belows are *r* and *P* values (NS: not significant).

	CD4	CD8	CD19	CD16-56	CD4/CD8	IgA (g/l)	IgM (g/l)	IgG (g/l)	Lymphocyte

Albumin (g/dl)	0.40	NS	NS	NS	NS	−0.41	NS	NS	0.40
	(.004)					(.003)			(.003)
Kt/V	NS	NS	NS	NS	NS	NS	NS	NS	NS
BMI (kg/m^2^)	NS	NS	NS	NS	NS	NS	NS	−0.32	NS
								(.03)	
Iron (μg/dl)	NS	NS	NS	NS	NS	NS	NS	NS	NS
Ferritin (ng/ml)	NS	−0.37	−0.54	NS	NS	NS	NS	NS	−0.28
		(.04)	(.002)						(.048)
Hemoglobin (g/dl)	NS	0.05	NS	NS	−0.28	NS	NS	NS	NS
		(.73)			(.05)				
Urea (mg/dl)	NS	0.04	NS	NS	NS	NS	NS	NS	NS
		(.77)							
Cholesterol (mg/dl)	0.35	0.38	0.29	NS	NS	NS	NS	NS	0.45
	(.01)	(.006)	(.04)						(.001)
Triglyceride (mg/dl)	0.34	0.41	NS	NS	NS	NS	NS	NS	0.41
	(.01)	(.003)							(.002)

## References

[1] (1998). Causes of death. United States Renal Data System. *American Journal of Kidney Diseases*.

[2] Raska K, Raskova J, Shea SM (1983). T cell subsets and cellular immunity in end-stage renal disease. *The American Journal of Medicine*.

[3] Kimmel PL, Phillips TM, Phillips E, Bosch JP (1990). Effect of renal replacement therapy on cellular cytokine
production in patients with renal disease. *Kidney International*.

[4] Chertow GM, Johansen KL, Lew N, Lazarus JM, Lowrie EG (2000). Vintage, nutritional status, and survival in hemodialysis patients. *Kidney International*.

[5] Rocco MV, Paranandi L, Burrowes JD (2002). Nutritional status in the HEMO study cohort at baseline. Hemodialysis. *American Journal of Kidney Diseases*.

[6] Weller I, Roitt I, Brostoff J, Male DK (2001). Secondary immunodeficiency. *Immunology*.

[7] McMurray DN, Loomis SA, Casazza LJ, Rey H, Miranda R (1981). Development of impaired cell-mediated immunity in mild
and moderate malnutrition. *The American Journal of Clinical Nutrition*.

[8] Wolfson M, Strong CJ, Minturn D, Gray DK, Kopple JD (1984). Nutritional status and lymphocyte function in maintenance
hemodialysis patients. *The American Journal of Clinical Nutrition*.

[9] Pupim LB, Caglar K, Hakim RM, Shyr Y, Ikizler TA (2004). Uremic malnutrition is a predictor of death independent
of inflammatory status. *Kidney International*.

[10] Haag-Weber M, Dumann H, Horl WH (1992). Effect of malnutrition and uremia on impaired cellular host defence. *Mineral and Electrolyte Metabolism*.

[11] Cancarini G, Costantino E, Brunori G (1992). Nutritional status in long-term CAPD patients. *Advances in Peritoneal Dialysis*.

[12] Kopple JD (1994). Effect of nutrition on morbidity and mortality in
maintenance dialysis patients. *American Journal of Kidney Diseases*.

[13] Chandra RK (1991). 1990 McCollum Award lecture.
Nutrition and immunity: lessons from the past and new
insights into the future. *The American Journal of Clinical Nutrition*.

[14] Iseki K, Yamazato M, Tozawa M, Takishita S (2002). Hypocholesterolemia is a significant predictor of death
in a cohort of chronic hemodialysis patients. *Kidney International*.

[15] Fujikawa M, Yamashita N, Yamazaki K, Sugiyama E, Suzuki H, Hamazaki T (1992). Eicosapentaenoic acid inhibits antigen-presenting
cell function of murine splenocytes. *Immunology*.

[16] Hughes DA, Pinder AC (2000). n-3 Polyunsaturated fatty acids inhibit the
antigen-presenting function of human monocytes. *The American Journal of Clinical Nutrition*.

[17] Muscaritoli M, Fanfarillo F, Luzi G (2001). Impaired nutritional status in common variable
immunodeficiency patients correlates with reduced
levels of serum IgA and of circulating
CD4^+^ T lymphocytes. *European Journal of Clinical Investigation*.

[18] Walker EM, Walker SM (2000). Effects of iron overload on the immune system. *Annals of Clinical and Laboratory Science*.

